# Persistent pulmonary congestion before discharge predicts rehospitalization in heart failure: a lung ultrasound study

**DOI:** 10.1186/s12947-015-0033-4

**Published:** 2015-09-04

**Authors:** Luna Gargani, P. S. Pang, F. Frassi, M.H. Miglioranza, F. L. Dini, P. Landi, E. Picano

**Affiliations:** National Research Council of Pisa, Institute of Clinical Physiology, Via G. Moruzzi, 1, 56124 Pisa, Italy; Department of Emergency Medicine, Indiana University School of Medicine, Indianapolis, USA; Department of Emergency Medicine, Azienda Ospedaliero-Universitaria, Pisa, Italy; Cardiology of Rio Grande do Sul, Porto Alegre, Brazil; Cardiac, Vascular and Thoracic Department, Azienda Ospedaliero-Universitaria, Pisa, Italy

**Keywords:** Lung ultrasound, B-lines, Ultrasound lung comets, Pulmonary congestion, Prognosis, Rehospitalization

## Abstract

**Background:**

B-lines evaluated by lung ultrasound (LUS) are the sonographic sign of pulmonary congestion, a major predictor of morbidity and mortality in patients with heart failure (HF). Our aim was to assess the prognostic value of B-lines at discharge to predict rehospitalization at 6 months in patients with acute HF (AHF).

**Methods:**

A prospective cohort of 100 patients admitted to a Cardiology Department for dyspnea and/or clinical suspicion of AHF were enrolled (mean age 70 ± 11 years). B-lines were evaluated at admission and before discharge. Subjects were followed-up for 6-months after discharge.

**Results:**

Mean B-lines at admission was 48 ± 48 with a statistically significant reduction before discharge (20 ± 23, *p* < .0001). During follow-up, 14 patients were rehospitalized for decompensated HF. The 6-month event-free survival was highest in patients with less B-lines (≤ 15) and lowest in patients with more B-lines (> 15) (log rank χ^2^ 20.5, *p* < .0001). On multivariable analysis, B-lines > 15 before discharge (hazard ratio [HR] 11.74; 95 % confidence interval [CI] 1.30–106.16) was an independent predictor of events at 6 months.

**Conclusions:**

Persistent pulmonary congestion before discharge evaluated by ultrasound strongly predicts rehospitalization for HF at 6-months. Absence or a mild degree of B-lines identify a subgroup at extremely low risk to be readmitted for HF decompensation.

## Introduction

Heart failure (HF) afflicts 1–2 % of people in the western world, with an incidence of 5–10 per 1000 persons per year [1]. Acute heart failure (AHF) is the most common reason for hospitalization in patients aged > 65 years [2, 3]. Despite significant improvement in signs and symptoms during hospitalization, post-discharge outcomes for patients hospitalized for AHF are poor. Up to 25 % of AHF patients are readmitted within 30 days of discharge, with a high mortality rate during this period [4].

Pulmonary congestion is a major predictor of morbidity and mortality in HF [5]. It is the single most important contributor to hospitalization, more significant than a low cardiac output [6]. Congestion is often not adequately addressed during hospitalization; patients experience improved symptoms, and may be free of clinical congestion but have persistent hemodynamic or pulmonary congestion [5, 7–9]. Failure to adequately relieve congestion during hospitalization is associated with increased morbidity and mortality, whereas patients discharged without congestion show better outcomes [10, 11].

Lung ultrasound (LUS) is a simple, accurate, and patient-friendly tool to assess pulmonary congestion, by evaluation of B-lines (previously called ultrasound lung comets) [12, 13]. B-lines are the sonographic sign of the pulmonary interstitial syndrome, representing the pulmonary interstitial edema in patients with AHF. Many studies have shown their utility in the differential diagnosis of acute dyspnea [14–20]. As such, assessment of B lines is now recommended in the pre-hospital and hospital management of AHF, as a bedside tool to enable direct visualization of interstitial edema in patients with suspected AHF [21]. Fewer studies are available on the prognostic role of pulmonary congestion assessed by B-lines, especially at discharge.

The aim of our study was to assess the prognostic significance of pulmonary congestion at discharge, as assessed by LUS B-lines, in patients admitted to a Cardiology Department with a suspicion of AHF. The study hypothesis is that in patients with detectable LUS at discharge the rate of events during follow-up is higher.

## Methods

### Patient population

A prospective cohort of 118 patients admitted to a Cardiology Department at a single center were enrolled. The inclusion criterion was the presence of signs and/or symptoms of AHF, irrespective of the etiology. Exclusion criteria were: 1) Inability to provide informed consent; 2) Known history of pulmonary fibrosis, pneumothorax, fibrothorax, lung cancer; 3) Patients not discharged home, but transferred to another department. From the initial population, 5 patients were excluded: 2 for known pulmonary fibrosis (1 patient with systemic sclerosis and 1 patient with post-irradiation thoracic fibrosis), 1 for fibrothorax, 1 for lung cancer, and 1 because she was transferred to another department and not discharged home.

The local Ethical Committee approved the protocol and all patients gave informed consent. The study conforms to the principles outlined in the Declaration of Helsinki.

### Lung ultrasound

Experienced sonographers (cardiologists and technicians accredited in transthoracic echocardiography by the European Association of Cardiovascular Imaging) performed the LUS examinations. LUS was performed twice in all patients: the first evaluation was done at admission at the end of standard echocardiography. The second evaluation was performed before discharge at the end of echocardiography, in case a second echocardiography was requested by the attending physicians, or as stand-alone examination. Patients were in the supine or near-to-supine position. The ultrasound scanning of the anterior and lateral chest was obtained as previously described on the right and left hemithoraxes. A detailed video showing how to perform a LUS examination to detect B-lines can be freely accessed at the following link: http://www.youtube.com/watch?v=amsULL ws8GI. A B-line was defined as a discrete laser-like vertical hyperechoic reverberation artifact that arises from the pleural line, extends to the bottom of the screen without fading, and moves synchronously with lung sliding [12]. In each intercostal space, the number of B-lines was recorded, and the sum of B-lines of each scanning sites yielded a score denoting the extent of the pulmonary interstitial edema. Zero was defined as a complete absence of B-lines in a scanning site. The presence of B-lines was staged in three grades, according to previous literature [22]: mild (a total of 6–15 B-lines on all scanning sites), moderate (a total of 16–30 B-lines on all scanning sites), and severe (>  30 B-lines on all scanning sites). The LUS examinations were performed with the same probe and same setting routinely used for echocardiographic studies. The intra- and interobserver variability of B-lines quantification have been previously tested in our laboratory as 5.1 and 7.4 %, respectively [23, 24]. A high reproducibility has been reported by other studies [25, 26].

### Reference standard definition of heart failure

Two cardiologists, who were blinded to the results of LUS evaluation, reviewed all the available medical records pertaining to the patient, and made an independent assessment of the probability of the patient having HF. Confirmation of AHF was based on the Framingham criteria, with corroborative information including clinical diary, information on hospital course, response to diuretics and other drugs, haemodynamic monitoring, electrocardiograms, chest X-ray, echocardiography, natriuretic peptides, and results of subsequent cardiac testing, including nuclear medicine or magnetic resonance imaging. For patients with a diagnosis other than HF, confirmation was attempted using the following variables: normal chest X-ray (lack of heart enlargement and signs of pulmonary congestion), normal systolic and diastolic heart function by echocardiography, normal valve function at echocardiography, normal natriuretic peptides, hospital clinical course. In cases when the two cardiologists failed to agree on a diagnosis, a consensus was reached with a third expert.

### Follow-up data

Follow-up data were obtained from at least 1 of 4 sources: review of the patient's hospital record, personal communication with the patient’s physician and review of the patient’s chart, a telephone interview with the patient conducted by trained personnel, a staff physician visiting the patients at regular intervals in the out-patient clinic. According to study protocol, follow-up information were obtained at 6 months. By inclusion criteria, follow-up data were obtained in all patients. Events were defined as rehospitalization for AHF in the 6 months following discharge. The development or progression of HF was defined according to the same criteria used for the reference standard definition of HF at admission. Patients who died were censored at the time of death.

### Statistical analysis

Continuous variables are expressed as mean ± SD. Two-sample comparisons were performed using t-test if variables were normally distributed, the Mann–Whitney U test for not normally distributed data, and the chi-squared test for categorical data. Death rates were estimated with Kaplan-Meier curves and compared by the log-rank test. A sample size calculation established a number of 91 patients to get an alpha error of 0.05, and a power of 0.9. The association of selected variables with outcome was assessed with the Cox’s proportional hazard model using univariable and stepwise multivariable procedures. A significance of p<0.05 was required for a variable to be included into the multivariable model, whilst p<0.1 was the cut-off value for exclusion. The following covariates were analysed at multivariable analysis: NYHA class, haemoglobin, NT-proBNP at discharge, B-lines at admission and at discharge. Hazard ratios (HR) with the corresponding 95 % confidence interval (CI) were estimated. Statistical significance was set at *p* < 0.05. All analyses were conducted with the Statistical Package for the Social Sciences (SPSS Inc., Chicago, Illinois, version 20) and GraphPad Prism version 6 (GraphPad Software Inc., San Diego, CA, USA).

## Results

### Cardiopulmonary ultrasound

Nine patients were excluded, after blinded reclassification, because of lack of signs and symptoms of HF. One patient was excluded because of in-hospital death. Three patients were lost at follow-up. Clinical characteristics of the final study population of 100 patients are shown in Table 1 and cardiac and lung ultrasound data are shown in Table 2. Ejection fraction was < 50 % in 74 patients; a significant diastolic dysfunction with restrictive pattern was present in 23 patients; 15 patients had a severe mitral regurgitation, 1 patient had a severe mitral stenosis, 3 patients had a severe aortic stenosis and 1 patient had a severe aortic regurgitation. The main aetiology of HF was coronary artery disease (52 patients), dilated cardiomyopathy (18 patients), valvular heart disease (15 patients), hypertensive heart disease (6 patients), arrhythmias (5 patients), hypertrophic cardiomyopathy (3 patients), chemotherapy-related cardiomyopathy (1 patient).

**Table 1:** Clinical characteristics of the study population

Patients *n* = 100	
Male (n, %)	73 (73 %)
Age (years)	70 ± 11
NYHA class	2.9 ± 0.9
SAP (mmHg)	126 ± 24
Diabetes Mellitus (n, %)	39 (39 %)
Hypertension (n, %)	57 (57 %)
Dyslipidemia (n, %)	39 (39 %)
Current smokers (n,%)	12 (12 %)
Creatinine (mg/dl)	1.3 ± 0.6
Hemoglobin (g/dl)	12.9 ± 2.1
CRP (mg/l)	1.4 ± 2
NT-proBNP (ng/l)	5291 ± 5877

*NYHA* New York Heart Association, *SAP* systolic arterial pressure, *CRP* C-reactive protein

**Table 2:** Cardiac and pulmonary ultrasound data of the study population

Ejection fraction (%)	37 ± 14
Wall motion score index	1.8 ± 0.56
LV end-diastolic diameter (mm)	58 ± 10
Impaired relaxation (n, %)	20 (20 %)
Pseudonormal pattern (n, %)	11 (11 %)
Restrictive pattern (n, %)	23 (23 %)
Moderate to severe mitral valve disease (n, %)	54 (54 %)
Moderate to severe aortic valve disease (n, %)	12 (12 %)
TAPSE (mm)	16.5 ± 4.7
PASP (mmHg)	49 ± 15
Pericardial effusion (n, %)	9 (9 %)
Pleural effusion (n, %)	24 (24 %)
B-lines at admission	48 ± 48
B-lines before discharge	20 ± 23

*LV* left ventricular, *TAPSE* tricuspid annular plane systolic excursion, *PASP* pulmonary artery systolic pressure

**Fig 1:**
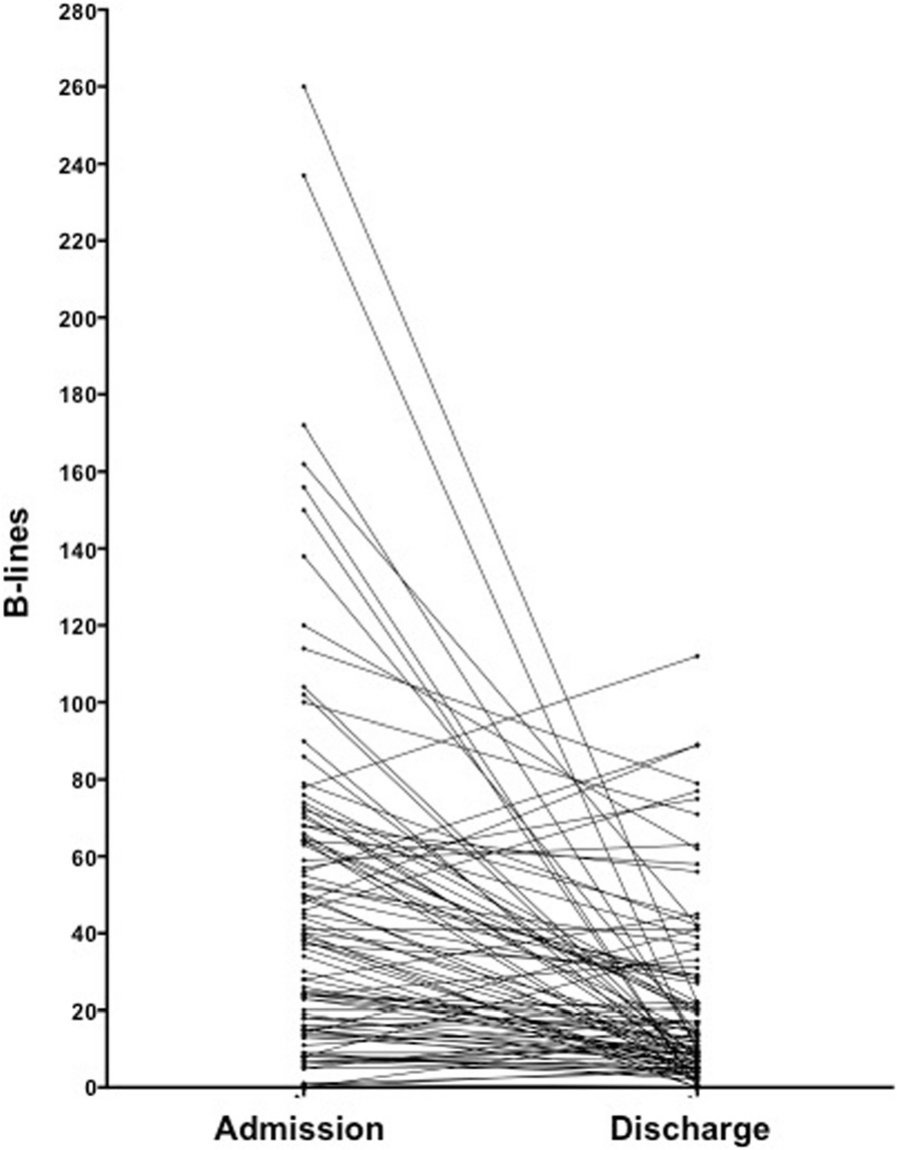
Individuals dynamic changes in admission-to-discharge B-lines

LUS scanning was feasible in 100 % of the cases and time needed for the examination was always less than 5 min. Mean B-lines at admission was 48 ± 48 with a statistically significant reduction before discharge (20 ± 23, *p* < .0001, Fig. 1). In 27 % of patients with a severe degree of B-lines at admission, the attending physician did not report pulmonary crackles at physical examination.

### Outcomes

The patient who died during hospitalization had a severe degree of B-lines in the last LUS scanning, the same day of his death. This patient was excluded from the analysis. During the 6-month follow-up a total of 14 rehospitalization for HF and 4 deaths occurred. Mean follow-up was 159 ± 50 days.

**Fig 2:**
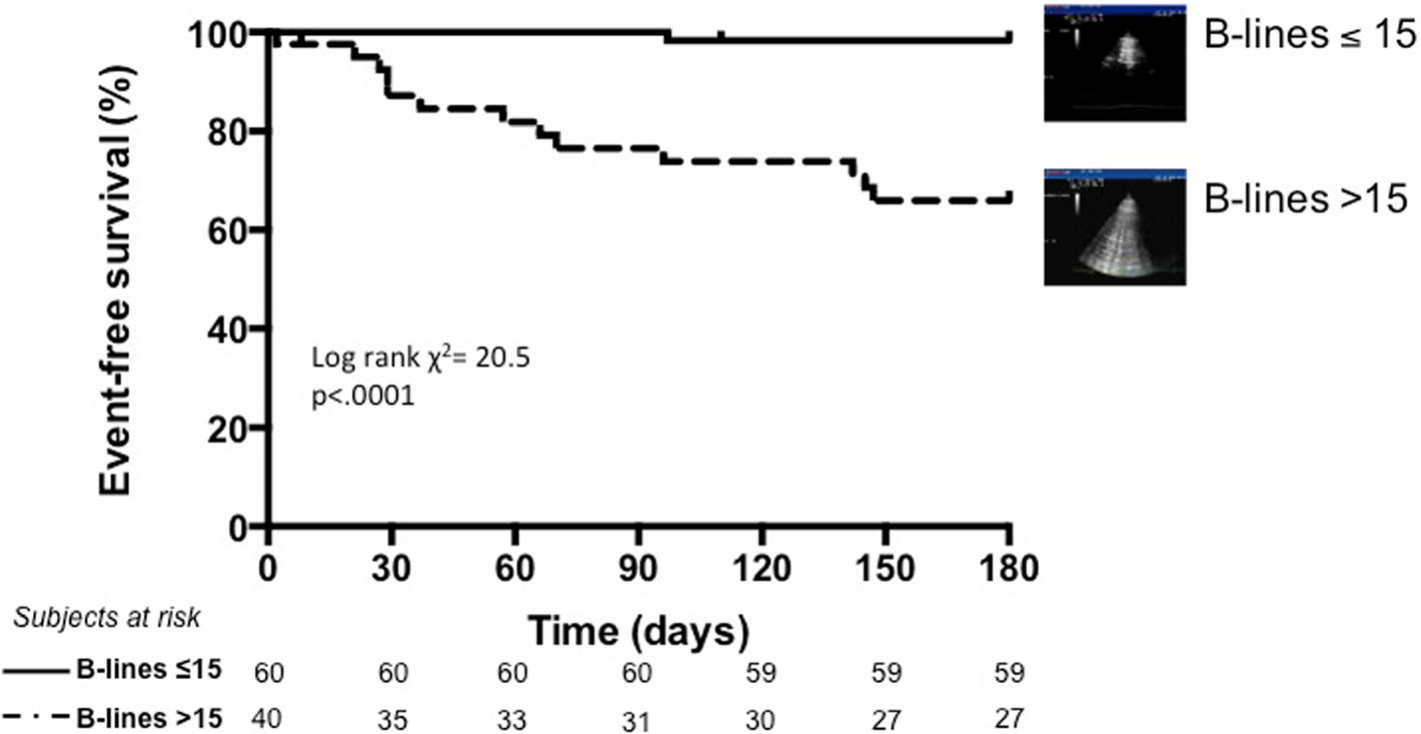
Kaplan-Meier survival curves in HF patients stratified according to the number of B-lines before discharge, at 6-months follow-up

A ROC analysis was performed to identify > 50 B-lines at admission as the best cut-off to predict readmission for HF during follow-up with a sensitivity of 71.4 %, specificity of 69.8 % negative predictive value of 93.7 %, positive predictive value of 27.8 %, negative likelihood ratio of 0.41, positive likelihood ratio of 2.36 (area under the curve 0.71, 95 % confidence interval 0.58–0.85, *p* = .011). A ROC analysis was performed to identify > 15 B-lines at discharge as the best cut-off to predict readmission for HF during follow-up with a sensitivity of 92.9 %, specificity of 68.6 % negative predictive value of 98.3 %, positive predictive value of 32.5 %, negative likelihood ratio of 0.1, positive likelihood ratio of 2.96 (area under the curve 0.83, 95 % confidence interval 0.74–0.92, *p* < .0001). The 6-month event-free survival showed a significantly better outcome for those patients with ≤ 15 B-lines at discharge, whereas a worse outcome was observed in patients with > 15 B-lines at discharge (log rank χ^2^ 20.5, *p* < .0001) (Fig. 2). Table 3 shows the parameters that were significantly different between patients with and without events. Predictors of HF hospitalization by univariate and multivariate analysis are reported in Table 4. At multivariable analysis, only a number of B-lines > 15 before discharge was an independent predictor of HF readmission at 6 months (hazard ratio [HR] 11.74; 95 % confidence interval [CI] 1.30–106.16).

**Table 3:** Different parameters in patients with and without events (HF readmission at 6 months)

	No HF readmission (*n* = 86)	HF readmission (*n* = 14)	p
Male sex	62 (72 %)	11 (79 %)	0.61
Age (years)	70.3 ± 10.9	69.8 ± 0.51	0.87
NYHA class	2.7 ± 0.9	3.4 ± 0.9	0.02
SAP (mmHg)	126 ± 22	124 ± 140	0.79
Diabetes Mellitus (n, %)	31 (36 %)	8 (57 %)	0.14
Hypertension (n, %)	49 (58 %)	8 (57 %)	0.37
Creatinine (mg/dl)	1.3 ± 0.5	1.6 ± 1.2	0.09
Hemoglobin (g/dl)	13.0 ± 2.1	12.0 ± 2.4	0.10
CRP (mg/l)	1.4 ± 2.1	1.1 ± 1.3	0.56
NT-proBNP (ng/l) at admission	5091 ± 5929	6203 ± 5821	0.57
NT-proBNP (ng/l) at discharge	3065 ± 4103	3816 ± 2764	0.09
Ejection fraction (%)	37 ± 13	37 ± 19	0.99
Wall motion score index	1.85 ± 0.57	1.83 ± 0.68	0.88
Restrictive pattern (n, %)	22 (26 %)	1 (7 %)	0.25
TAPSE (mm)	16.8 ± 4.7	14.5 ± 4.2	0.14
PASP (mmHg)	48 ± 14	54 ± 15	0.20
Pericardial effusion (n, %)	7 (8 %)	2 (14 %)	0.51
Pleural effusion (n, %)	19 (23 %)	5 (36 %)	0.42
B-lines at admission	43 ± 43	79 ± 66	0.01
B-lines at discharge	17 ± 22	40 ± 24	<0.0001

*NYHA* New York Heart Association, *SAP* systolic arterial pressure, *CRP* C-reactive protein, *TAPSE* tricuspid annular plane systolic excursion, *PASP* pulmonary artery systolic pressure

**Table 4:** Univariate and multivariate analysis to predict events (HF readmission at 6 months)

Univariate analysis			Multivariate analysis	
	HR (95 % CI)	p	HR (95 % CI)	p
Age ^a^	0.99 (0.95 – 1.04)	0.88		
NYHA class ^a^	2.31 (1.16 – 4.60)	0.017	1.48 (0.63 – 3.49)	0.37
Diabetes Mellitus	2.19 (0.76 – 6.31)	0.15		
Creatinine ^a^	1.58 (0.90 – 2.76)	0.11		
Hemoglobin ^a^	0.77 (0.59 – 1.02)	0.07		
Hemoglobin < 10 g/dl	5.75 (1.76 – 18.78)	0.004	3.96 (0.52 – 30.10)	0.52
NT-proBNP at admission ^a^	1.01 (0.99 – 1.01)	0.54		
NT-proBNP at discharge ^a^	1.01 (0.99 – 1.01)	0.55		
NT-proBNP at discharge > 1635 ng/l	10.65 (1.35 – 84.26)	0.025	3.98 (0.43 – 37.18)	0.23
Ejection fraction ^a^	1.01 (0.96 – 1.04)	0.91		
Wall motion score index ^a^	0.88 (0.35 – 2.17)	0.77		
Restrictive pattern	0.33 (0.04 – 2.72)	0.30		
TAPSE ^a^	0.91 (0.80 – 1.03)	0.13		
PASP ^a^	1.03 (0.99 – 1.07)	0.11		
Pericardial effusion (n, %)	1.68 (0.38 – 7.51)	0.49		
Pleural effusion (n, %)	1.67 (0.51 – 5.49)	0.39		
B-lines at admission ^a^	1.01 (1.01 – 1.02)	0.004		
B-lines at admission > 50	5.83 (1.82 – 18.62)	0.003	4.87 (0.88 – 27.06)	0.07
B-lines at discharge ^a^	1.03 (1.01 – 1.04)	0.001		
B-lines at discharge > 15	24.12 (3.15 – 184.55)	0.002	11.74 (1.30 – 106.16)	0.028

*NYHA* New York Heart Association, *TAPSE* tricuspid annular plane systolic excursion, *PASP* pulmonary artery systolic pressure, *HR* hazard ratio, *CI* confidence interval

a = continuous variable

**Fig 3:**
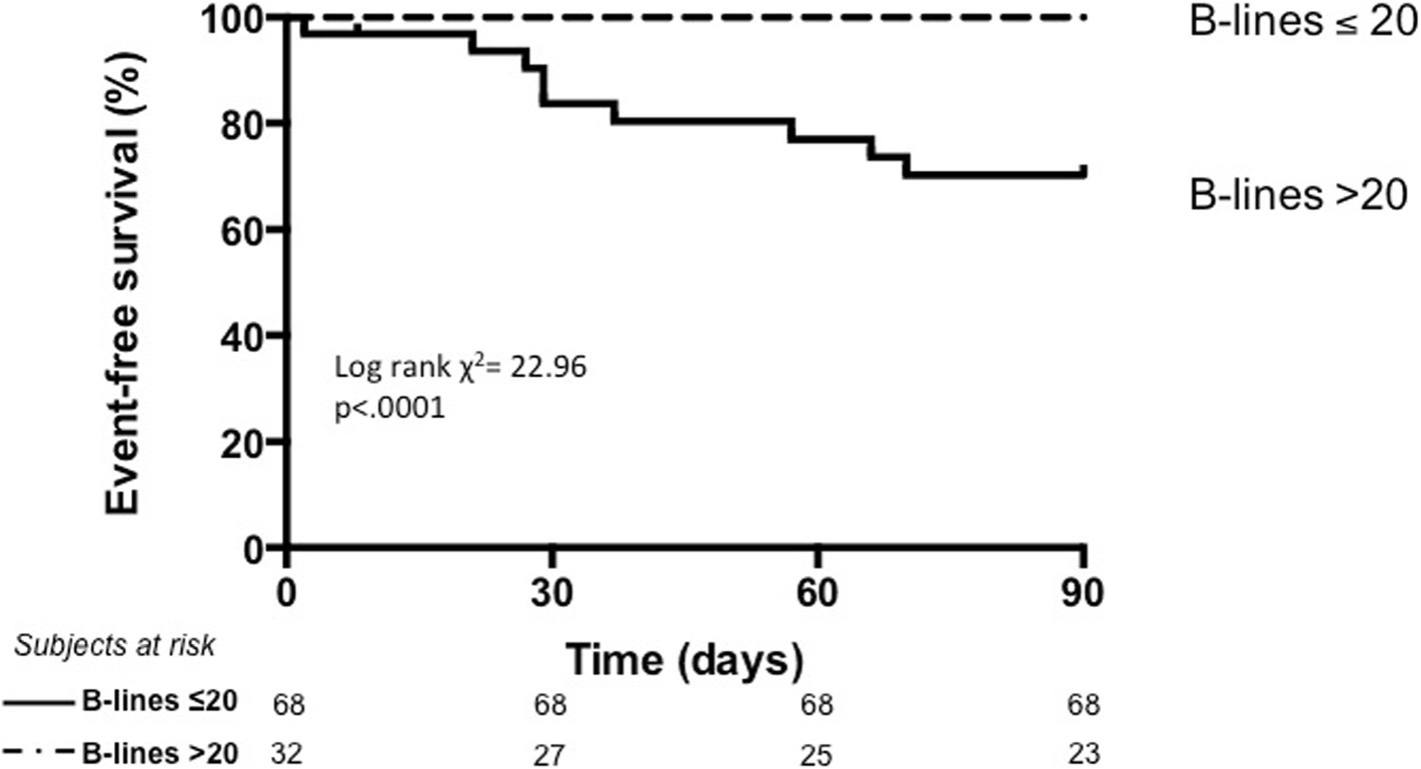
Kaplan-Meier survival curves in HF patients stratified according to the number of B-lines before discharge, at 3-months follow-up

When considering a follow-up of 3 months, the same cut-off of > 15 B-lines predicted readmission for HF with a sensitivity of 100 % and specificity of 64.8 %. The ROC analysis identified > 20 B-lines at discharge as the best cut-off to predict readmission for HF at 3 months follow-up, with a sensitivity of 100 %, a specificity of 74.7 %, negative predictive value of 100 %, positive predictive value of 28.1 %, negative likelihood ratio of 0, positive likelihood ratio of 3.96 (area under the curve 0.88, 95 % confidence interval 0.81-0.96, *p* < .0001). Kaplan-Meier curves depicting the prognostic value of > 20 B-lines before discharge at a 3 months follow-up are shown in Fig. 3 (log rank χ^2^ 22.96, *p* < .0001).

## Discussion

In patients admitted with AHF to a cardiology department, the absence of significant pulmonary congestion before discharge, assessed by lung ultrasound, identifies a subgroup at a very low risk of being readmitted for decompensated HF in the following 6 months.

### Pathophysiology of persistent congestion

The role of persistent congestion at hospital discharge is relevant in the outcome of patients with AHF. The early post-discharge time interval immediately after hospitalization, which is often addressed to as the *vulnerable phase* [27], has been recognized as a crucial moment in the management of AHF, when the majority of events occur [28, 29]. Patients with HF die or are hospitalized for different reasons, including ischemia, arrhythmias, worsened hemodynamics. However, in the vast majority the underlying pathophysiology is primarily related to increasing left ventricular filling pressures [30]. In the continuum of the congestion cascade in HF, it is important to distinguish the different phases of congestion [5, 31]: increased left ventricular filling pressures represent a phase of hemodynamic congestion, which is different from pulmonary, and from systemic and clinical congestion. In this cascade, pulmonary congestion more specifically refers to extravascular lung water (EVLW). Patients with the same degree of LV filling pressures may have significantly different degrees of pulmonary congestion, from complete absence of EVLW to alveolar pulmonary edema. These differences rely on many aspects, which are often difficult to be assessed, including the integrity of alveolar-capillary membrane – that depends also on the length of the disease -, the systemic inflammation status which may influence vascular permeability, and lymphatic drainage. LUS evaluation of B-lines offers a specific visualization of pulmonary congestion [32], thus easily allowing a non-invasive and bedside assessment of the degree of EVLW in any single patient. This information adds to the assessment of hemodynamic congestion that can be non-invasively performed through echoDoppler parameters. Natriuretic peptides, which are very useful at all steps of the management of HF patients, reflect more hemodynamic than pulmonary congestion. This can explain why natriuretic peptides and B-lines are somehow correlated in relatively large populations [15], but can provide very different information in the single patient, one enhancing the value of the other, especially in patients with natriuretic peptides in the grey zone [16, 17].

It is worth highlighting that there is no standardized method to assess pulmonary congestion in the clinical arena [5]. Chest X-ray is the most widely used tool to establish the presence and degree of EVLW, allowing a visualization of the lungs in the context of the whole chest. However, both sensitivity and specificity to detect signs of pulmonary congestion are suboptimal. Nearly 20 % of patients with HF have a normal chest X-ray, limiting overall sensitivity [21, 33].

### Comparison with previous studies

The search for an optimal tool to predict rehospitalization for HF has been going on for years. Some scores have been proposed, although predictive models of HF hospitalization are still not currently implemented in the clinical practice. Most scores have been validated on chronic HF in ambulatory outpatients, and include clinical, biochemical and sometimes instrumental data, such as the HFPSI (Heart Failure Patient Severity Index) [34], the CHARM score [35], and more recently the REDIN score [36].

Previous studies have demonstrated the prognostic role of B-lines as an independent predictor of events in patients admitted with dyspnea and/or chest pain, in acute coronary syndromes, in hemodialysis, and in chronic HF even when assessed by a hand-held ultrasound device [37–41]. However, these analysis refer to admission exams and not to discharge, when the clinical information derived from a timely assessment can be relevant not only in a population-based approach, but also in a patient-based risk assessment, useful for management decisions, including discharge therapy, diuretic dosage, follow-up timing.

### Clinical implications

In the last few years the use of B-lines in the management of patient with acute and chronic HF has moved from the research setting to the clinical arena, and its assessment is now included in Recommendation papers [21, 42, 43].

Our data shows that patients with persistent sonographic pulmonary congestion have an increased risk for rehospitalization for HF in the next 6 months, but the main finding of our study is the very low risk of readmission in patients with ≤ 15 B-lines before discharge. Thus, assessment of B-lines would allow for tailored discharge risk stratification. While further studies are needed, persistent presence of B-lines may be useful to titrate therapy as well as to personalize discharge planning and timing of follow-up.

It is interesting to note that although both B-lines at admission and at discharge are predictors of rehospitalization at univariate analysis, B-lines at discharge have a higher hazard ratio and are the only independent predictors of events at multivariate analysis. The best cut-offs obtained by ROC analysis to maximize accuracy are also different: at admission a higher number of B-lines is needed to confer an increased risk of events at 6-months, compared to discharge. This is reasonable, given the dynamic behavior of B-lines that can be highly responsive to treatment [44], and may rapidly change even after the short time of a dialysis session [25, 45], or increase significantly during the few minutes of a stress-echo test [46].

The possibility to count B-lines has been debated in the past. B-lines are substantially rough ultrasound artifacts, but by now many studies have shown the good correlations between the somehow “imprecise” number of B-lines and more established parameters of increased EVLW and decompensation [15, 17, 24, 25, 47–52]. In the clinical routine, however, the eyeballing imaging of B-lines (the whiter the image under the pleural line, the more B-lines) can be enough to get quick but meaningful information on the degree of pulmonary congestion. Moreover, the possibility to implement specific software which may aid in establishing the severity of B-lines, would further simplify the process [53].

We found > 15 and > 20 B-lines to be the best cut-offs to maximize accuracy in the prediction of HF readmission at 6 and 3 months. This cut-offs, although derived from independent statistical analysis, are consistent with previous studies showing that ≥ 15 B lines predicts decompensation in outpatients with HF [54]. It is worth noting however, that counting B-lines should not be equated to a precise assessment such as dosing a metabolite, and a difference in a few B-lines is not of clinical importance. The spatial distribution of B-lines and not only the absolute number of B-lines should be taken into account: first, it is known that a few B-lines, especially at pulmonary bases, can be found in normal subjects [13]; then, a very small number of B-lines found in many different scanning sites, although yielding an absolute high total number, may be less clinically relevant than many B-lines in a smaller number of scanning sites. As with any other test, B-lines should not be interpreted in isolation, but integrated with the overall clinical picture.

Despite not being a sophisticated, high tech parameter, this simple ultrasound biomarker seems to effectively help in the management of patients with HF, in a very sustainable and patient-friendly approach.

### Limitations

This is a single-centre study with a relatively small study group of patients with HF of different aetiologies. This limitation however reflects the potentialities of LUS B-lines in different clinical scenarios, including patients with HF due to acute coronary syndromes. Given the relatively small study population, the number of events is also low. However, the number of patients enrolled was determined as sufficient according to sample size calculation. It should be emphasized that B-lines are a non-specific sign of pulmonary interstitial syndrome, which can be found also in interstitial pulmonary fibrosis, interstitial pneumonia and acute respiratory distress syndrome. This is crucial especially when using B-lines for the differential diagnosis of dyspnoea. When B-lines are used to determine persistent pulmonary congestion in patients with an already established diagnosis of HF, this limitation is less relevant. Interpreting B-lines not as a single image, but in a specific clinical context, is the key to avoid gross misinterpretation of this sign. When presence or persistence of B-lines is totally unrelated to the clinical picture, caution should be used and other causes of B-lines should be taken into account (pulmonary fibrosis in patients on amiodarone, non cardiogenic pulmonary edema, interstitial lung disease). Persistent pulmonary congestion can be related to the dosage of pharmacological therapy, especially diuretics; unfortunately, we did not add the specific dosage of therapies administered during hospital stay in our study population. Our findings should be confirmed in a larger multicentre study.

## Conclusions

In AHF patients, persistent pulmonary congestion as defined by ≥ 15 B lines strongly predicts rehospitalization in the following 6-months. Absence or a mild degree of B-lines identify a subgroup at extremely low risk to be readmitted for AHF. Sonographic B-lines can be useful not only for the differential diagnosis of acute dyspnea, but also for the prognostic stratification of HF patients.

## Abbreviations

AHF: Acute heart failure
HF: Heart failure
LUS: Lung ultrasound
LV: Left ventricle
EVLW: Extra-vascular lung water

## References

[1] Mosterd A, Hoes AW (2007). Clinical epidemiology of heart failure. Heart.

[2] Yancy CW, Jessup M, Bozkurt B, Butler J, Casey DE, Drazner MH, Fonarow GC, Geraci SA, Horwich T, Januzzi JL, Johnson MR, Kasper EK, Levy WC, Masoudi FA, McBride PE, McMurray JJ V, Mitchell JE, Peterson PN, Riegel B, Sam F, Stevenson LW, Tang WHW, Tsai EJ, Wilkoff BL (2013). ACCF/AHA guideline for the management of heart failure: A report of the american college of cardiology foundation/american heart association task force on practice guidelines. Circulation.

[3] McMurray JJ V, Adamopoulos S, Anker SD, Auricchio A, Böhm M, Dickstein K, Falk V, Filippatos G, Fonseca C, Gomez-Sanchez MA, Jaarsma T, Køber L, Lip GYH, Pietro MA, Parkhomenko A, Pieske BM, Popescu BA, Rønnevik PK, Rutten FH, Schwitter J, Seferovic P, Stepinska J, Trindade PT, Voors AA, Zannad F, Zeiher A (2013). ESC guidelines for the diagnosis and treatment of acute and chronic heart failure 2012: the task force for the diagnosis and treatment of acute and chronic heart failure 2012 of the european society of cardiology. Developed in collaboration with the heart. Rev Port Cardiol.

[4] Gheorghiade M, Vaduganathan M, Fonarow GC, Bonow RO (2013). Rehospitalization for heart failure. J Am Coll Cardiol.

[5] Gheorghiade M, Follath F, Ponikowski P, Barsuk JH, Blair JEA, Cleland JG, Dickstein K, Drazner MH, Fonarow GC, Jaarsma T, Jondeau G, Sendon JL, Mebazaa A, Metra M, Nieminen M, Pang PS, Seferovic P, Stevenson LW, Van Veldhuisen DJ, Zannad F, Anker SD, Rhodes A, McMurray JJV, Filippatos G (2010). Assessing and grading congestion in acute heart failure: a scientific statement from the acute heart failure committee of the heart failure association of the European society of cardiology and endorsed by the European society of intensive care medicine. Eur J Heart Fail.

[6] Gheorghiade M, Abraham WT, Albert NM, Greenberg BH, O’Connor CM, She L (2006). SYstolic blood pressure at admission, clinical characteristics, and outcomes in patients hospitalized with acute heart failure. JAMA.

[7] Gheorghiade M, Pang P, Ambrosy A, Lan G, Schmidt P, Filippatos G, Konstam M, Swedberg K, Cook T, Traver B, Maggioni A, Burnett J, Grinfeld L, Udelson J, Zannad F (2012). A comprehensive, longitudinal description of the in-hospital and post-discharge clinical, laboratory, and neurohormonal course of patients with heart failure who die or are re-hospitalized within 90 days: analysis from the EVEREST trial. Heart Fail Rev.

[8] Picano E, Gargani L, Gheorghiade M (2010). Why, when, and how to assess pulmonary congestion in heart failure: pathophysiological, clinical, and methodological implications. Heart Fail Rev.

[9] Gheorghiade M, Shah AN, Vaduganathan M, Butler J, Bonow RO, Rosano GMC, Taylor S, Kupfer S, Misselwitz F, Sharma A, Fonarow GC (2013). Recognizing hospitalized heart failure as an entity and developing new therapies to improve outcomes: academics’, clinicians’, industry’s, regulators’, and payers’ perspectives. Heart Fail Clin.

[10] Gattis WA, O’Connor CM, Gallup DS, Hasselblad V, Gheorghiade M (2004). Predischarge initiation of carvedilol in patients hospitalized for decompensated heart failure: results of the initiation management predischarge: process for assessment of carvedilol therapy in heart failure (IMPACT-HF) trial. J Am Coll Cardiol.

[11] Investigators TE, Coordinators ES (2005). Evaluation study of congestive heart failure and pulmonary artery catheterization effectiveness: The escape trial. JAMA.

[12] Volpicelli G, Elbarbary M, Blaivas M, Lichtenstein DA, Mathis G, Kirkpatrick AW, Melniker L, Gargani L, Noble VE, Via G (2012). International Liaison Committee on Lung Ultrasound (ILC-LUS) for Interna tional Consensus Conference on Lung Ultrasound (ICC-LUS). International evidence-based recommendations for point-of-care lung ultrasound. Intensive Care Med.

[13] Gargani L (2011). Lung ultrasound: a new tool for the cardiologist. Cardiovasc Ultrasound.

[14] Lichtenstein D, Mezière G (1998). A lung ultrasound sign allowing bedside distinction between pulmonary edema and COPD: the comet-tail artifact. Intensive Care Med.

[15] Gargani L, Frassi F, Soldati G, Tesorio P, Gheorghiade M, Picano E (2008). Ultrasound lung comets for the differential diagnosis of acute cardiogenic dyspnoea: a comparison with natriuretic peptides. Eur J Heart Fail.

[16] Prosen G, Klemen P, Štrnad M, Grmec S (2011). Combination of lung ultrasound (a comet-tail sign) and N-terminal pro-brain natriuretic peptide in differentiating acute heart failure from chronic obstructive pulmonary disease and asthma as cause of acute dyspnea in prehospital emergency setting. Crit Care.

[17] Liteplo AS, Marill KA, Villen T, Miller RM, Murray AF, Croft PE, Capp R, Noble VE (2009). Emergency thoracic ultrasound in the differentiation of the etiology of shortness of breath (ETUDES): sonographic B-lines and N-terminal pro-brain-type natriuretic peptide in diagnosing congestive heart failure. Acad Emerg Med.

[18] Cibinel GA, Casoli G, Elia F, Padoan M, Pivetta E, Lupia E, Goffi A (2012). Diagnostic accuracy and reproducibility of pleural and lung ultrasound in discriminating cardiogenic causes of acute dyspnea in the Emergency Department. Intern Emerg Med.

[19] Ricci F, Aquilani R, Radico F, Bianco F, Dipace GG, Miniero E, De Caterina R, Gallina S (2015). Role and importance of ultrasound lung comets in acute cardiac care. Eur Hear J Acute Cardiovasc Care.

[20] Pivetta E, Goffi A, Lupia E, Tizzani M, Porrino G, Ferreri E, et al. LUng ultrasound-implemented diagnosis of acute decompensated heart failure in the emergency department - a simeu multicenter study. Chest. 2015;148(1):202–10. 10.1378/chest.14-2608 25654562

[21] Mebazaa A, Yilmaz MB, Levy P, Ponikowski P, Peacock WF, Laribi S et al. Recommendations on pre-hospital and early hospital management of acute heart failure: a consensus paper from the Heart Failure Association of the European Society of Cardiology, the European Society of Emergency Medicine and the Society of Academic Emergency Medicine. Eur Heart J. 2015 Aug 7;36(30):1958–66. 10.1093/eurheartj/ehv066 25998514

[22] Picano E, Frassi F, Gligorova S, Gargani L, Mottola G, Agricola E (2006). Ultrasound lung comets: a clinically useful sign of extravascular lung water. J Am Soc Echocardiogr.

[23] Bedetti G, Gargani L, Corbisiero A, Frassi F, Poggianti E, Mottola G (2006). Evaluation of ultrasound lung comets by hand-held echocardiography. Cardiovasc Ultrasound.

[24] Jambrik Z, Monti S, Coppola V, Agricola E, Mottola G, Miniati M, Picano E (2004). Usefulness of ultrasound lung comets as a nonradiologic sign of extravascular lung water. Am J Cardiol.

[25] Mallamaci F, Benedetto FA, Tripepi R, Rastelli S, Castellino P, Tripepi G, Picano E, Zoccali C (2010). Detection of pulmonary congestion by chest ultrasound in dialysis patients. JACC Cardiovasc Imaging.

[26] Basso F, Milan Manani S, Cruz DN, Teixeira C, Brendolan A, Nalesso F, Zanella M, Ronco C (2013). Comparison and reproducibility of techniques for fluid status assessment in chronic hemodialysis patients. Cardiorenal Med.

[27] Greene SJ, Fonarow GC, Vaduganathan M, Khan SS, Butler J, Gheorghiade M (2015). The vulnerable phase after hospitalization for heart failure. Nat Rev Cardiol.

[28] Adams KF, Fonarow GC, Emerman CL, LeJemtel TH, Costanzo MR, Abraham WT, Berkowitz RL, Galvao M, Horton DP (2005). Characteristics and outcomes of patients hospitalized for heart failure in the United States: Rationale, design, and preliminary observations from the first 100,000 cases in the Acute Decompensated Heart Failure National Registry (ADHERE). Am Heart J.

[29] Desai AS, Stevenson LW (2012). Rehospitalization for heart failure: Predict or prevent?. Circulation.

[30] Zile MR, Bennett TD, St. John Sutton M, Cho YK, Adamson PB, Aaron MF, Aranda JM, Abraham WT, Smart FW, Stevenson LW, Kueffer FJ, Bourge RC (2008). Transition from chronic compensated to acute d compensated heart failure: Pathophysiological insights obtained from continuous monitoring of intracardiac pressures. Circulation.

[31] Gheorghiade M, Filippatos G, De Luca L, Burnett J (2015). Congestion in acute heart failure syndromes: an essential target of evaluation and treatment. Am J Med.

[32] Volpicelli G, Skurzak S, Boero E, Carpinteri G, Tengattini M, Stefanone V, Luberto L, Anile A, Cerutti E, Radeschi G, Frascisco MF (2014). Lung ultrasound predicts well extravascular lung water but is of limited usefulness in the prediction of wedge pressure. Anesthesiology.

[33] Collins SP, Lindsell CJ, Storrow AB, Abraham WT (2006). Prevalence of negative chest radiography results in the emergency department patient with decompensated heart failure. Ann Emerg Med.

[34] Hummel SL, Ghalib HH, Ratz D, Koelling TM (2013). Risk stratification for death and all-cause hospitalization in heart failure clinic outpatients. Am Heart J.

[35] Pocock SJ, Wang D, Pfeffer MA, Yusuf S, McMurray JJ V, Swedberg KB, Ostergren J, Michelson EL, Pieper KS, Granger CB (2006). Predictors of mortality and morbidity in patients with chronic heart failure. Eur Heart J.

[36] Álvarez-García J, Ferrero-Gregori A, Puig T, Vázquez R, Delgado J, Pascual-Figal D, Alonso-Pulpón L, González-Juanatey JR, Rivera M, Worner F, Bardají A, Cinca J. Investigators of the Spanish Heart Failure Network (REDINSCOR). A simple validated method for predicting the risk of hospitalization for worsening of heart failure in ambulatory patients: the Redin-SCORE. Eur J Heart Fail. 2015;17(8):818–27. 10.1002/ejhf.287 PMC5032982 26011392

[37] Zoccali C, Torino C, Tripepi R, Tripepi G, D’Arrigo G, Postorino M, Gargani L, Sicari R, Picano E, Mallamaci F, D’Arrigo G (2013). Pulmonary congestion predicts cardiac events and mortality in ESRD. J Am Soc Nephrol.

[38] Siriopol D, Hogas S, Voroneanu L, Onofriescu M, Apetrii M, Oleniuc M, Moscalu M, Sascau R, Covic A (2013). Predicting mortality in haemodialysis patients: A comparison between lung ultrasonography, bioimpedance data and echocardiography parameters. Nephrol Dial Transplant.

[39] Gustafsson M, Alehagen U, Johansson P. Imaging Congestion With a Pocket Ultrasound Device: Prognostic Implications in Patients With Chronic Heart Failure. J Card Fail. 2015;21(7):548–54. 10.1016/j.cardfail.2015.02.004 25725475

[40] Frassi F, Gargani L, Tesorio P, Raciti M, Mottola G, Picano E (2007). Prognostic value of extravascular lung water assessed with ultrasound lung comets by chest sonography in patients with dyspnea and/or chest pain. J Card Fail.

[41] Bedetti G, Gargani L, Sicari R, Gianfaldoni ML, Molinaro S, Picano E (2010). Comparison of prognostic value of echocardiacgraphic risk score with the Thrombolysis in Myocardial Infarction (TIMI) and Global Registry in Acute Coronary Events (GRACE) risk scores in acute coronary syndrome. Am J Cardiol.

[42] Neskovic AN, Hagendorff A, Lancellotti P, Guarracino F, Varga A, Cosyns B, Flachskampf FA, Popescu BA, Gargani L, Zamorano JL (2013). Emergency echocardiography: the European Association of Cardiovascular Imaging recommendations. Eur Hear J Cardiovascular Imaging.

[43] Lancellotti P, Price S, Edvardsen T, Cosyns B, Neskovic AN, Dulgheru R, Flachskampf FA, Hassager C, Pasquet A, Gargani L, Galderisi M, Cardim N, Haugaa KH, Ancion A, Zamorano J-L, Donal E, Bueno H, Habib G (2015). The use of echocardiography in acute cardiovascular care: Recommendations of the European Association of Cardiovascular Imaging and the Acute Cardiovascular Care Association. Eur Hear J Acute Cardiovasc Care.

[44] Volpicelli G, Melniker LA, Cardinale L, Lamorte A, Frascisco MF (2013). Lung ultrasound in diagnosing and monitoring pulmonary interstitial fluid. Radiol Med.

[45] Noble VE, Murray AF, Capp R, Sylvia-Reardon MH, Steele DJR, Liteplo A (2009). Ultrasound assessment for extravascular lung water in patients undergoing hemodialysis. Time course for resolution. Chest.

[46] Agricola E, Picano E, Oppizzi M, Pisani M, Meris A, Fragasso G, Margonato A (2006). Assessment of stress-induced pulmonary interstitial edema by chest ultrasound during exercise echocardiography and its correlation with left ventricular function. J Am Soc Echocardiogr.

[47] Jambrik Z, Gargani L, Adamicza Á, Kaszaki J, Varga A, Forster T, Boros M, Picano E, Adamicza A (2010). B-lines quantify the lung water content: a lung ultrasound versus lung gravimetry study in acute lung injury. Ultrasound Med Biol.

[48] Agricola E, Bove T, Oppizzi M, Marino G, Zangrillo A, Margonato A, Picano E (2005). “Ultrasound comet-tail images”: a marker of pulmonary edema: a comparative study with wedge pressure and extravascular lung water. Chest.

[49] Frassi F, Gargani L, Gligorova S, Ciampi Q, Mottola G, Picano E (2007). Clinical and echocardiographic determinants of ultrasound lung comets. Eur J Echocardiogr.

[50] Donadio C, Bozzoli L, Colombini E, Pisanu G, Ricchiuti G, Picano E, Gargani L (2015). Effective and timely evaluation of pulmonary congestion: qualitative comparison between lung ultrasound and thoracic bioelectrical impedance in maintenance hemodialysis patients. Medicine (Baltimore).

[51] Gargani L, Picano E, Caramella D, Abramo A, Giunta F, Forfori F, Baldi G, D’Errico L (2013). Lung water assessment by lung ultrasonography in intensive care: a pilot study. Intensive Care Med.

[52] Pratali L, Cavana M, Sicari R, Picano E (2010). Frequent subclinical high-altitude pulmonary edema detected by chest sonography as ultrasound lung comets in recreational climbers. Crit Care Med.

[53] Raso R, Gargani L, Tartarisco G, Pioggia G, Picano E (2012). Operator-independent soft computing-based assessment of extravascular lung water by ultrasound. European Heart Journal. Volume 33.

[54] Miglioranza MH, Gargani L, Sant’anna RT, Rover MM, Martins VM, Mantovani A, Weber C, Moraes MA, Feldman CJ, Kalil RAK, Sicari R, Picano E, Leiria TLL (2013). Lung ultrasound for the evaluation of pulmonary congestion in outpatients: a comparison with clinical assessment, natriuretic peptides, and echocardiography. JACC Cardiovasc Imaging.

